# Increased plasma levels of epithelial neutrophil-activating peptide 78/CXCL5 during the remission of Neuromyelitis optica

**DOI:** 10.1186/s12883-016-0622-3

**Published:** 2016-07-11

**Authors:** Tao Yang, Su Wang, Qi Zheng, Lei Wang, Qian Li, Mingyan Wei, Zongpan Du, Yongping Fan

**Affiliations:** Department of Traditional Chinese Medicine, Beijing Tiantan Hospital, Capital Medical University, Beijing, 100050 People’s Republic of China; Department of Oncology, Hiser Medical Center of Qingdao, Qingdao, 266034 People’s Republic of China; Department of oncology, Guang An Men Hospital of China Academy of Chinese Medical Sciences, Beijing, 100053 People’s Republic of China; School of Traditional Chinese Medicine, Capital Medical University, Beijing, 100050 People’s Republic of China

**Keywords:** Neuromyelitis optica, Epithelial neutrophil-activating peptide 78, Interleukin 1β, Tumor necrosis factor α

## Abstract

**Background:**

In neuromyelitis optica (NMO), one of the underlying pathogenic mechanisms is the formation of antigen-antibody complexes which can trigger an inflammatory response by inducing the infiltration of neutrophils in lesions. Epithelial neutrophil-activating peptide 78 (ENA 78), known as Chemokine (C-X-C motif) ligand 5 (CXCL5), belongs to the ELR-CXCL family. It recruits and activates neutrophils. The aim of this study was to evaluate ENA 78, IL-1β and TNF-α plasma levels in multiple sclerosis (MS) and neuromyelitis optica (NMO) patients.

**Methods:**

ENA 78, IL-1β and TNF-α plasma levels were detected in 20 healthy controls (HC), 25 MS and 25 NMO patients using MILLIPLEX® map Human High Sensitivity Cytokine/Chemokine Panels.

**Results:**

Plasma levels of ENA 78 were significantly higher in NMO patients than in HC (*P* < 0.001) and MS patients (*P* < 0.05). The NMO patients showed higher plasma levels of IL-1β compared with HC (*P* < 0.01). Further, increased plasma levels of TNF-α were found in the MS (*P* < 0.05) and NMO patients (*P* < 0.001). In addition, NMO patients had higher Expanded Disability Status Scale (EDSS) scores compared with MS patients (*P* < 0.05). EDSS scores were correlated with plasma levels of ENA 78 in NMO patients (*P* < 0.05). There were no significant correlations between EDSS scores and plasma levels of ENA 78 in MS patients (*P* > 0.05).

**Conclusions:**

The overproduction of pro-inflammatory cytokines such as IL-1β and TNF-α during the remission of NMO activates ENA 78, which in turn leads to neutrophil infiltration in lesions. ENA 78 plasma levels were correlated with EDSS scores in NMO patients. Elevated secretion of ENA 78 may be a critical step in neutrophil recruitment during the remission of NMO.

## Background

Neuromyelitis optica (NMO, Devic’s syndrome) and multiple sclerosis (MS) are autoimmune and degenerative diseases characterized by demyelination of central nervous system (CNS), potentially leading to paralysis and other clinical symptoms [1–4]. NMO and MS are two of the most common diseases causing neurological disability in young adults [1, 5, 6]. Accumulating evidence has shown that NMO pathogenesis differs from MS, including aquaporin 4 (AQP4)-IgG increase and infiltration of granulocytes and macrophages [7, 8].

A significant feature distinguishing NMO from MS is the relatively higher number of neutrophils, eosinophils, macrophages and fewer T cells in the lesions [2, 8, 9]. Abnormal neutrophil aggregation in the lesions and increased AQP4-IgG are the notable features distinguishing NMO from MS [8, 10]. Neutrophil protease inhibition reduces AQP4-IgG damage in the mouse brain, which suggests that neutrophils play an important role in NMO pathology [7]. Studies also suggest a tight regulation of neutrophils and immune cell recruitment in NMO [2]. The innate immune response is orchestrated by inflammatory cells as a cascade of events, and each stage is associated with inflammatory cell recruitment and infiltration. Neutrophil infiltration is triggered by epithelial neutrophil-activating peptide 78 (ENA 78), which plays a role in tissue repair, metabolism, microbial killing, and angiogenesis [11, 12]. ENA 78 is a member of CXC chemokines, which enhance leukocyte recruitment and activation in autoimmune disorders and inflammatory diseases [13–15]. Its aberrant expression has been detected in rheumatoid arthritis, psoriasis, autism, bacterial meningitis, etc. [16–20]. ENA 78 is categorized into sub-classes based on the sequence and function, and characterized by the ELR (glutamic acid-leucine-arginine) motif preceding the N-terminal Cys and activating C-X-C chemokine receptor type (CXCR) 2 selectively [21]. ENA 78 as a potent ELR^+^ CXC chemokine attracts and activates polymorphonuclear leukocytes (PMNLs) which are higher in patients with infections, inasmuch as the PMNLs are among the first cells to exist the peripheral blood and migrate to the inflammatory site [16, 22, 23].

This study was the first to evaluate plasma levels of ENA 78 and its relation to Expanded Disability Status Scale (EDSS) scores in NMO patients.

## Methods

### Study populations

Written informed consent was obtained from all participants. The study protocol was approved by the local ethics committee (IRB of Beijing Tiantan Hospital Affiliated to Capital Medical University, No. KY2015-003-02). MS and NMO patients were recruited from Beijing Tiantan Hospital, Capital Medical University. This study was conducted between May and July 2015 on 20 healthy controls (HC), 25 patients with MS and 25 patients with NMO. Plasma samples were obtained from MS patients, NMO patients and 20 HC recruited from the general population without immune diseases. Infections were ruled out by full blood count in all subjects.

The MS diagnosis was determined according to the 2010 revised McDonald criteria [24] and the NMO diagnosis was based on the revised diagnostic criteria for NMO [25]. The interviews, neurological examinations and EDSS scores of the MS and NMO groups were conducted in an MS cohort study. All the NMO patients had optica neuritis and myelitis and met at least 2 of the 3 supporting criteria (Brain MRI-, AQP4-IgG+, negative for MS).

### Plasma chemokine and cytokines levels

To exclude the effect of different time points and other factors on the level of chemokines and cytokines, all blood samples (2 mL each) were obtained at 9:00 a.m using disposable Ethylenediaminetetraacetic acid (EDTA) vacuum blood collection tubes (BD, USA) and tested over 8 h. After 2 h of standing at 4 °C, the supernatant was pipetted into EP tubes and stored at −80 °C. Plasma ENA 78, IL-1β and TNF-α were measured using MILLIPLEX® map Human High Sensitivity Cytokine/Chemokine Panels (Cat. No. HCYP2MAG-62 K; Cat. No. HCYTOMAG-60 K), according to the manufacturer’s instructions.

### Statistical analysis

Statistical analysis was performed using GraphPad Prism version 5 (GraphPad Software, Inc., California) and the data were reported as Means ± SEM. Mann–Whitney U and Kruskal-Wallis tests were used to compare 2 to 3 groups, respectively. Pearson’s test was used to perform correlations. A *P* value of < 0.05 was considered statistically significant.

## Results

### Clinical demographics

The subjects included 20 HC, 25 MS and 25 NMO patients. No significant age differences were found in the three groups. Age of onset, disease duration and annual relapse rate (ARR) of MS and NMO patients also showed no significant differences. All the subjects were aged between 10 and 60 years. There was a significant difference between the MS and NMO groups in EDSS scores (*P* < 0.05) (Table 1).

**Table 1:** Demographic and clinical data of HC, MS and NMO

		HC (*n* = 20)	MS (*n* = 25)	NMO (*n* = 25)
Gender	F/M	15/5	19/6	23/2
Age (year)	Range	25–54	16–59	14–55
	Mean ± SE	32.30 ± 1.80	33.84 ± 2.31	36.80 ± 2.45
Age at onset (year)	Range	-	6–57	13–53
	Mean ± SE	-	29.84 ± 2.28	30.24 ± 2.34
Disease duration (year)	Range	-	0–12	0–21
	Mean ± SE	-	3.92 ± 0.53	6.04 ± 1.01
ARR	Range	-	0.35–12.00	0.01–12.00
	Mean ± SE	-	1.61 ± 0.47	1.69 ± 0.47
EDSS	Range	-	0–3.5	1–6.5
	Mean ± SE	-	1.92 ± 0.28	3.24 ± 0.35*

*HC* healthy controls, *MS* multiple sclerosis, *NMO* Neuromyelitis optica, *EDSS* expanded disability status scale, *ARR* annual relapse rate

*P < 0.05

### Increased plasma ENA 78, IL-1β and TNF-α levels in NMO patients

**Fig. 1:**
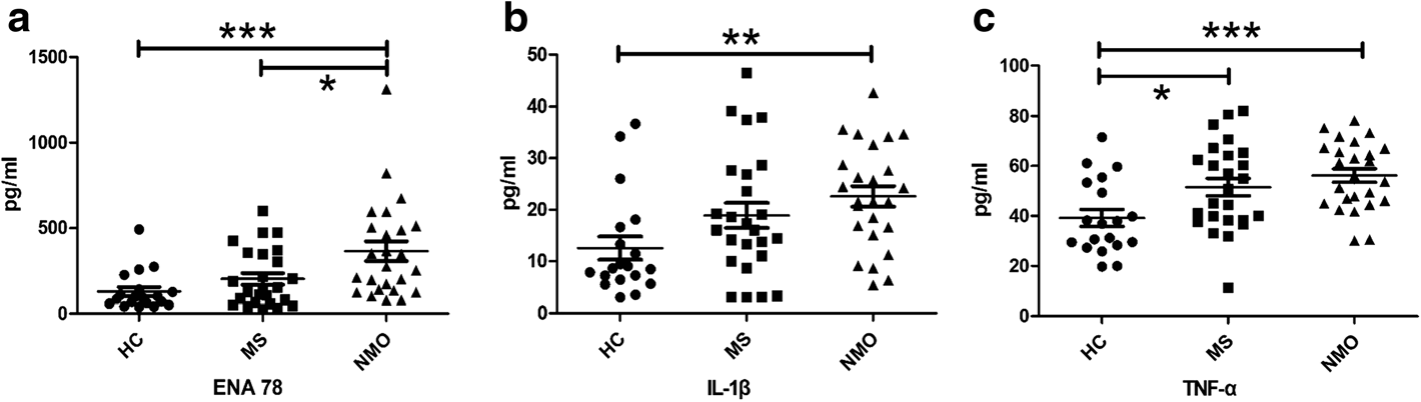
Comparison of chemokine and cytokine levels among the HC, MS and NMO groups. HC = healthy controls; MS = multiple sclerosis; NMO = neuromyelitis optica. **a** Comparison of plasma ENA 78 levels among the HC, MS and NMO. **b** Comparison of plasma IL-1β level among the HC, MS and NMO. **c** Comparison of plasma TNF-α level among the HC, MS and NMO. *P < 0.05, **P < 0.01, ***P < 0.001

ENA 78 expression was higher in NMO plasma than in HC (*P* < 0.001) and MS (*P* < 0.05) (Fig. 1a). The cytokine IL-1β potentially induces ENA 78 secretion. As shown in (Fig. 1b), the IL-1β level in NMO was higher than in HC (*P* < 0.01) with no significant difference between MS and HC, MS and NMO. The cytokine TNF-α levels were higher in NMO than in HC (*P* < 0.001) (Fig. 1c).

### Correlation between ENA 78 gradients and EDSS scores in the MS and NMO groups

**Fig. 2:**
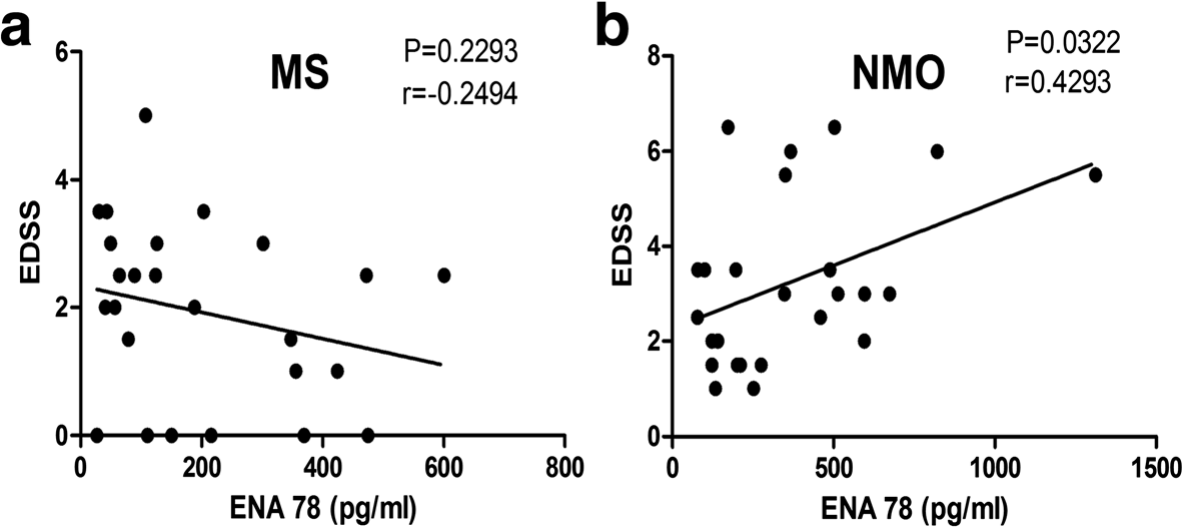
Each data point represents an individual subject. **a** Correlation between ENA 78 and EDSS scores in the MS. **b** Correlation between ENA 78 and EDSS scores in the NMO

ENA 78 plasma levels were not correlated with EDSS scores in MS (Fig. 2a). Significant correlation existed between ENA 78 gradients and EDSS scores in NMO (*P* < 0.05) (Fig. 2b).

## Discussion

The perivascular presence of neutrophils is one of the primary histological differences between MS and NMO, as reported in NMO patients as well as in mouse and rat models [2, 7, 26]. Neutrophils are elevated about 20 % in the CSF during remission of NMO patients [27]. In mouse models of NMO, tissue damage was ameliorated by eliminating neutrophils, whereas increased neutrophils exacerbated tissue damage [7, 28]. ENA 78 is one of the ELR^+^ chemokines specifically inducing neutrophil migration, with the ability to interact with chemokine receptors CXCR1 and CXCR2 [29, 30]. ENA 78 stimulates neutrophil directed chemotaxis by promoting the intracellular level of elastase and free calcium and inducing neutrophil pro-adhesive activity [31, 32]. In addition, inhibitors of neutrophil elastase, which are involved in neutrophil migration and neutrophil-mediated tissue damage, have been tested in experimental trials such as Sivelestat [7, 33–35]. Other studies also indicated that the increased ENA 78 amplified the pro-inflammatory cytokine response, which had a direct chemo-attracting effect on neutrophils [36–39]. Therefore, we studied ENA 78 and found that it was dramatically increased in the NMO patients (vs. HC, *P* < 0.001; vs. MS, *P* < 0.05). Studies proved that ENA 78 was detected in eosinophils, which also aggregated in the NMO lesions [2, 8, 40], suggesting that eosinophils recruit and activate CXCR2^+^ cells such as neutrophils by secreting ENA 78. In the present study, we found that the plasma ENA 78 gradient was correlated with EDSS in NMO patients rather than in MS (*P* < 0.05). ENA 78 causes neutrophil aggregation and hyperactivation around the lesions in NMO resulting in demyelination, which is different from the pathophysiological mechanisms of MS. The higher ENA 78 gradient in the blood leads to increased neutrophil aggregation around the lesions, causing severe clinical symptoms.

Although the precise mechanism involving ENA 78 upregulation is not fully understood, factors involved in modulating ENA 78 expression at the transcriptional level and signaling pathways are known in different types of cells [41–43]. Neutrophil chemoattractant chemokines belonging to ELR-CXCL family, especially ENA 78 binding with chemokine receptor CXCR2, mediate the IL-1β driven cell recruitment [43, 44]. ELR^+^ chemokines, including CXCL1, CXCL2 and ENA 78, are triggered by IL-1β [45–47]. IL-1β inducing ENA 78 expression by activating cAMP-response element binding protein (CREB) and NF-kB promoter of ENA 78 is part of the inflammatory response *in vitro* and *in vivo* [39, 43, 48–50]. IL-1β induces leukocyte rolling, adherence and emigration associated with an increase in kinin B1 receptor mRNA expression, which plays a role in neutrophil recruitment [44]. This current study results showed that increased IL-1β levels in NMO patients matched the higher ENA 78 levels in the periphery compared with the HC (*P* < 0.01). TNF-α, which induces neutrophil influx, exacerbates the lesions in *ex vivo* spinal cord and optical nerve of NMO [28, 51]. Our findings, herein, show that NMO and MS patients had higher plasma levels of TNF-α compared with HC (*P* < 0.001; *P* < 0.05, respectively). Further, TNF-α potentially increases the adhesion-molecule expression in the brain suggesting a role for anti-TNF therapies in NMO [8, 52, 53]. The overexpression of IL-1β and TNF-α might be one of the factors inducing severe lesions in NMO, exacerbating the damage mediated by higher ENA 78 levels.

## Conclusions

In summary, the overproduction of pro-inflammatory cytokines such as IL-1β and TNF-α during remission of NMO might result in activation of ENA 78. High levels of ENA 78 may play a critical role in neutrophil infiltration during NMO inflammation. And these might lead to increased neutrophils aggregation around the lesion, causing severer clinical symptoms once NMO relapse. ENA 78 plasma levels were also correlated with EDSS scores in NMO remission. The current study enables the therapy of NMO patients.

## Abbreviations

AQP, aquaporin; CREB, cAMP-response element binding protein; CNS, central nervous system; CXCL, chemokine (C-X-C motif) ligand; NMO, Neuromyelitis optica; NMO, neuromyelitis optica, ENA, epithelial neutrophil-activating peptide, EDTA, Ethylenediaminetetraacetic acid, MS, multiple sclerosis; HC, healthy controls; EDSS, expanded disability status score; IL-1β, interleukin 1β; TNF-α, tumor necrosis factor α; PMNLs, polymorphonuclear leukocytes; ELR, glutamic acid-leucine-arginine motif
